# Sensitivity Analysis of the Scattering-Based SARBM3D Despeckling Algorithm

**DOI:** 10.3390/s16070971

**Published:** 2016-06-25

**Authors:** Alessio Di Simone

**Affiliations:** Department of Electrical Engineering and Information Technology, University of Naples Federico II, Naples 80125, Italy; alessio.disimone@unina.it; Tel.: +39-081-768-3801

**Keywords:** Synthetic Aperture Radar (SAR), denoising, scattering, fractals, nonlocal means

## Abstract

Synthetic Aperture Radar (SAR) imagery greatly suffers from multiplicative speckle noise, typical of coherent image acquisition sensors, such as SAR systems. Therefore, a proper and accurate despeckling preprocessing step is almost mandatory to aid the interpretation and processing of SAR data by human users and computer algorithms, respectively. Very recently, a scattering-oriented version of the popular SAR Block-Matching 3D (SARBM3D) despeckling filter, named Scattering-Based (SB)-SARBM3D, was proposed. The new filter is based on the a priori knowledge of the local topography of the scene. In this paper, an experimental sensitivity analysis of the above-mentioned despeckling algorithm is carried out, and the main results are shown and discussed. In particular, the role of both electromagnetic and geometrical parameters of the surface and the impact of its scattering behavior are investigated. Furthermore, a comprehensive sensitivity analysis of the SB-SARBM3D filter against the Digital Elevation Model (DEM) resolution and the SAR image-DEM coregistration step is also provided. The sensitivity analysis shows a significant robustness of the algorithm against most of the surface parameters, while the DEM resolution plays a key role in the despeckling process. Furthermore, the SB-SARBM3D algorithm outperforms the original SARBM3D in the presence of the most realistic scattering behaviors of the surface. An actual scenario is also presented to assess the DEM role in real-life conditions.

## 1. Introduction

During the last two decades, an increasing interest in remotely-sensed data is evident within the international scientific community. The availability of more and more powerful hardware resources and accurate sensors explains the increasing number of applications of remotely-sensed data, spanning from precise agriculture to water source management, from urban area monitoring to celestial bodies analysis. Owing to their all-day and all-weather capabilities, Synthetic Aperture Radar (SAR) sensors are currently of great interest, as witnessed by the recent COSMO-SkyMed and TerraSAR-X missions and the ongoing Sentinel mission by the European Space Agency (ESA). However, the readability of SAR products is dramatically impaired by the multiplicative speckle noise, typical of coherent acquisition systems, like SAR sensors. Data interpretation by SAR-expert users and scene analysis by computer programs is almost impractical unless an accurate speckle reduction (despeckling) preprocessing step is performed. Since the pioneering works by Goodman [1], Lee [2,3], Frost [4] and Kuan [5], the despeckling problem has been of huge interest within the SAR community, as witnessed by the very extensive related literature. Up to now, numerous approaches and techniques to despeckling have been proposed [6,7,8,9,10,11]. Among these methods, the most promising ones can be arguably considered the nonlocal-means and wavelet-based approaches [10,11]. In the nonlocal means framework, a novel intensity-based metric is introduced in order to overcome the edge smearing effects typical of the average filters, based on pure geometrical distances. The despeckling process is carried out by averaging similar pixels, usually collected in patches, regardless of their geometrical distance. The nonlocal concept originally developed in [12] for additive noise was adapted to the peculiarities of speckle noise in SAR imagery in [10], in which the Probability Patch-Based (PPB) filter was derived within a Weighted Maximum Likelihood Estimation framework. More recently, the very promising SAR Block-Matching 3D (SARBM3D) algorithm was presented in [11]. It represents a SAR-oriented version of the block-matching 3D filter first proposed in [13] and suitable for additive noise, in which the most advanced image processing concepts, such as nonlocal means, wavelet analysis and collaborative filtering are properly merged in a two-step algorithm. Very recently, a novel physical-based approach to the despeckling problem was proposed [14]. The novel framework was applied to the PPB [15] and SARBM3D filters [16]. The new filters were named Scattering-Based (SB)-PPB and SB-SARBM3D, respectively. In [15,16], physical concepts are introduced via the estimation of the energy backscattered from the surface. The backscattered signal, estimated assuming the knowledge of the surface topography, is used as a kind of a priori information within the filters. Accordingly, a Digital Elevation Model (DEM) of the considered scene coregistered to the SAR image must be available to apply the proposed SB-PPB and SB-SARBM3D filters. The scattering-based techniques exhibit better performance w.r.t. the state of the art, both in terms of speckle reduction and detail preservation [15,16]. It is noticeable that, at least in principle, any despeckling algorithm coping with SAR intensity models could take advantage of scattering issues via the insertion of physical-based concepts into the filter.

In this paper, we provide an experimental sensitivity analysis of the SB-SARBM3D filter. In particular, the following features of the scene and of the algorithm are analyzed:

• the scattering model describing the surface;
• surface parameter errors apart from the local incidence angle;
• the DEM resolution (on both simulated and actual SAR data);
• errors in the SAR image-DEM coregistration step.

The paper is organized as follows. In Section 2, the original SARBM3D and the proposed scattering-based version are briefly described. In Section 3, the sensitivity analysis is described, and the main results are presented and discussed. Section 4 concludes the paper with some relevant remarks and future recommendations.

## 2. Materials and Methods

In this section, we briefly describe both SARBM3D and SB-SARBM3D for a full understanding of the following sensitivity analysis. After a conceptual overview of SARBM3D, we explore its scattering-based version in some more detail, introducing also the notation used in the following. For more information, the reader is referred to the original papers [11,16].

### 2.1. SARBM3D

The SARBM3D filter originally proposed in [11] is a SAR-oriented version of the block-matching 3D filter [13] suitable for the peculiarities of speckle noise affecting SAR imagery.

In [11], the multiplicative noise model is converted in the following additive signal-dependent noise model:

(1) z(s)=x(s)n(s)=x(s)+x(s)[n(s)−1]=x(s)+v(s)

where x(·) and n(·) stand for clean data and noise, respectively.

The despeckling process is carried out in a two-step algorithm in which several of the most advanced concepts in denoising—nonlocal filtering, block-matching, wavelet shrinkage—are introduced. In each step, three processing blocks are performed: block-matching, collaborative filtering and aggregation. In the first step, local image statistics are estimated via a collaborative nonlocal block-matching approach with a metrics suitable for the multiplicative speckle noise. In particular, the following distance is used to evaluate the similarity between geometrically close blocks in *L*-look SAR images:

(2) $$d\left[a\left(B_{s}\right),\;a\left(B_{t}\right)\right]=\;\left(2L\;-\;1\right)\underset{k}{\sum }\log\left[\frac{a\left(s\;+\;k\right)}{a\left(t\;+\;k\right)}+\frac{a\left(t\;+\;k\right)}{a\left(s\;+\;k\right)}\;\right]$$

where $B_{s}$ indicates a block centered on pixel *s*, $a\left(B_{s}\right)$ the corresponding amplitudes and *k* scans the block pixels. This distance has been used with success in several nonlocal despeckling techniques [10,11,15,16]. For each reference block, the most similar blocks are grouped in a 3D stack; a hard-thresholding in the wavelet domain performs the collaborative filtering. Local image statistics are estimated by means of an optimal linear minimum mean square error (MMSE) estimation framework. Under the constraint of linearity, the optimal MMSE estimator reads as:

(3) $${\hat{X}}_{1}=E\left[X\right]+\;\left(C_{X}\right){\left(C_{X}+C_{V}\right)}^{-1}\left(Z-E\left[Z\right]\right)$$

where the capital letters stands for the wavelet coefficients, boldface indicates the coefficients of the blocks grouped in vectors, E[·] denotes the statistical expectation, $C_{X}$ and $C_{V}$ represent the covariance matrices of X and V, respectively, and ${\hat{X}}_{1}$ is the first-step outcome. In Equation (3), uncorrelation between clean signal and noise is assumed. Then, by supposing the covariance matrices to be diagonal, applying the shrinkage only to the coefficients of the detail sub-bands and then resorting to some reasonable simplifications, the filtered wavelet coefficients reads as [11]:

(4) $${\hat{X}}_{1}\left(i\right)=\max\left(0,\;\frac{{\left\langle Z^{2}\right\rangle }_{SB\left(i\right)}-\frac{\sigma_{u}^{2}}{\left(1+\sigma_{u}^{2}\right)}{\left\langle Z^{2}\right\rangle }_{G}}{{\left\langle Z^{2}\right\rangle }_{SB\left(i\right)}}\right)Z\left(i\right)$$

where ${\left\langle \cdot \right\rangle }_{SB\left(i\right)}$ and ${\left\langle \cdot \right\rangle }_{G}$ stand for the average over the sub-band comprising the *i*-th coefficient and the whole group, respectively, and $\sigma_{u}^{2}$ is a known parameter depending on speckle format and number of looks [11]. In Equation (4), all quantities within the brackets ${\left\langle \cdot \right\rangle }_{SB\left(i\right)}$ and ${\left\langle \cdot \right\rangle }_{G}$ can be estimated reliably by sample averages over the undecimated discrete wavelet transform (UDWT) sub-band and over the whole 3D stack, respectively. The local image statistics are estimated from the outcome of the first step and used in the second one, where the actual despeckling is performed via a 3D collaborative Wiener filtering in the wavelet domain. Similarly, a linear MMSE approach is exploited for the collaborative filtering in the second step. The final estimate reads as:

(5) $${\hat{X}}_{2}\left(i\right)=\;\frac{{\hat{X}}_{1}^{2}\left(i\right)}{{\hat{X}}_{1}^{2}\left(i\right)+{\left\langle V^{2}\right\rangle }_{G}\;}Z\left(i\right)$$

### 2.2. SB-SARBM3D

The first step of SARBM3D provides a first estimate of the filtered image used as a pilot in the second step. A good pilot is essential for the success of the final despeckling step, especially when the original image is very noisy, as is the case of single-look SAR images. The better the pilot, the more reliable the estimates and the better the final outcome. In order to provide a better pilot image for *L*-look SAR images of natural scenes, in the SB-SARBM3D filter, the scattering behavior of the scene is exploited as a priori information in the first step of SARBM3D. The a priori scattering information is estimated from the underlying topography via a proper scattering model. In particular, the sensed scene is modeled via a (topological) 2D fractional Brownian motion (fBm) with Hurst coefficient *H* (0 ≤ *H* ≤1) and topothesy *T* [m]. The electromagnetic energy backscattered from the surface is derived under the Small Perturbation Method (SPM), according to which the backscattering coefficient of the surface $\sigma^{0}$ is related to both the surface and the sensor parameters as follows:

(6) $$\sigma_{mn}^{0}=2\pi 8k^{4}S_{0}{\left|\beta_{mn}\right|}^{2}\frac{\cos^{4}\vartheta }{{\left(2k\sin\vartheta \right)}^{2+2H}}$$

wherein *m* and *n* denote the transmitted and received polarizations, respectively, and may stand for horizontal or vertical polarization; *k* is the electromagnetic wavenumber of the incident field; *S_0_* is a parameter characterizing the spectral behavior of the physical fBm surface, expressed in [m^−2−2H^], and related to *T* and *H* [17]; $\beta_{mn}$, accounting for the incident and reflected fields’ polarization, is a function of both the relative dielectric constant $\varepsilon_{r}$, the electrical conductivity σ of the surface and the local incidence angle ϑ [17].

As shown in the Appendix in [15], the huge number of parameters influencing the signal backscattered from the surface does not prevent a satisfactory (for the speckle filtering purposes) estimation of the a priori scattering information in Equation (6), which can be provided once the knowledge of the most influencing parameter (i.e., the local incidence angle) is assumed. According to this approach, in [15,16], a DEM of the sensed surface is exploited to compute the local incidence angle map needed for the backscattering coefficient estimation. It is noteworthy that, in order to apply the SB-SARBM3D filter, a Digital Elevation Model (DEM) of the surface in azimuth-slant range and coregistered to the SAR image is required. Concerning the remaining parameters, namely *H*, *T*, $\varepsilon_{r}$ and σ, reference values for soil—$\varepsilon_{r}=4$, $\sigma =10^{-2}$ S/m, *H* = 0.8 and *T* = $10^{-5}$ m—are used as input if no a priori information is available, i.e., they are assumed constant over the entire scene. However, it is noteworthy that an angle-independent linear regression-based retrieval algorithm is described in [18,19] for the *H* map estimation from a single SAR image. This map can be used as input in the algorithm without a significant extra computational load.

To improve the quality of the pilot image, the scattering behavior of the surface is exploited in SB-SARBM3D via a proper averaging of the pilot image provided by the original SARBM3D algorithm ${\hat{x}}_{1,\;SARBM3D}$ and the a priori scattering information [16]. The new pilot image ${\hat{x}}_{1,\;SB-SARBM3D}$ reads as:

(7) $${\hat{x}}_{1,\;SB-SARBM3D}\left(s\right)=w\left(s\right){\hat{x}}_{1,\;SARBM3D}\left(s\right)+\left(1-w\left(s\right)\right){\hat{\sigma }}^{0}\left(s\right)$$

where ${\hat{\sigma }}^{0}$ is the backscattering coefficient estimated via Equation (6) assuming the availability of a scene DEM; the parameter w∈[0, 1] weights the two terms. The weight map is designed to account for the reliability of both terms, i.e., it is close to one (zero) where the first step of SARBM3D (the scattering model) is effective. The reliability of both terms is quite opposite, since the first-step estimation of SARBM3D is highly reliable in urban areas and in regions with non-topography-related SAR intensity variations, where the proposed single-bounce scattering model is not accurate due to the surface homogeneity assumption. On the contrary, the scattering model accurately describes the scattering behavior of both homogeneous areas and regions with topography-related SAR intensity variations, where the block-matching processing of SARBM3D causes some artifacts. Therefore, to define a sensible weight map, an identification of both non-topographic edges and man-made structures is needed beforehand. To this aim, the ratio detectors proposed by Lopes et al. [20,21] are applied to both the input SAR image (*r_I_*) and the local incidence angle map (*r_ϑ_*). The ratio maps *r_I_* and *r_ϑ_* are aimed at detecting peculiar features—lines, points, edges—in the SAR image and the local incidence angle map, respectively. Ratio maps values much lower than one reveal the presence of a feature. In order to properly weigh the pilot image provided by the original SARBM3D filter and the a priori scattering information, the weight map is evaluated as follows:

(8) $$w\left(s\right)=1-\min\left(\frac{r_{I}\left(s\right)}{r_{\vartheta }\left(s\right)},\frac{r_{\vartheta }\left(s\right)}{r_{I}\left(s\right)}\right)$$

Equation (8) maps topographic features in low weights, since they are detected in both maps, and non-topographic features in high weights, since they are correctly detected in the *r_I_* map only. Consequently, a high weight is assigned to the a priori scattering information in correspondence of topographic features; on the contrary, the filter assigns a high weight to the SARBM3D first step estimation in correspondence of non-topographic features.

**Figure 1:**
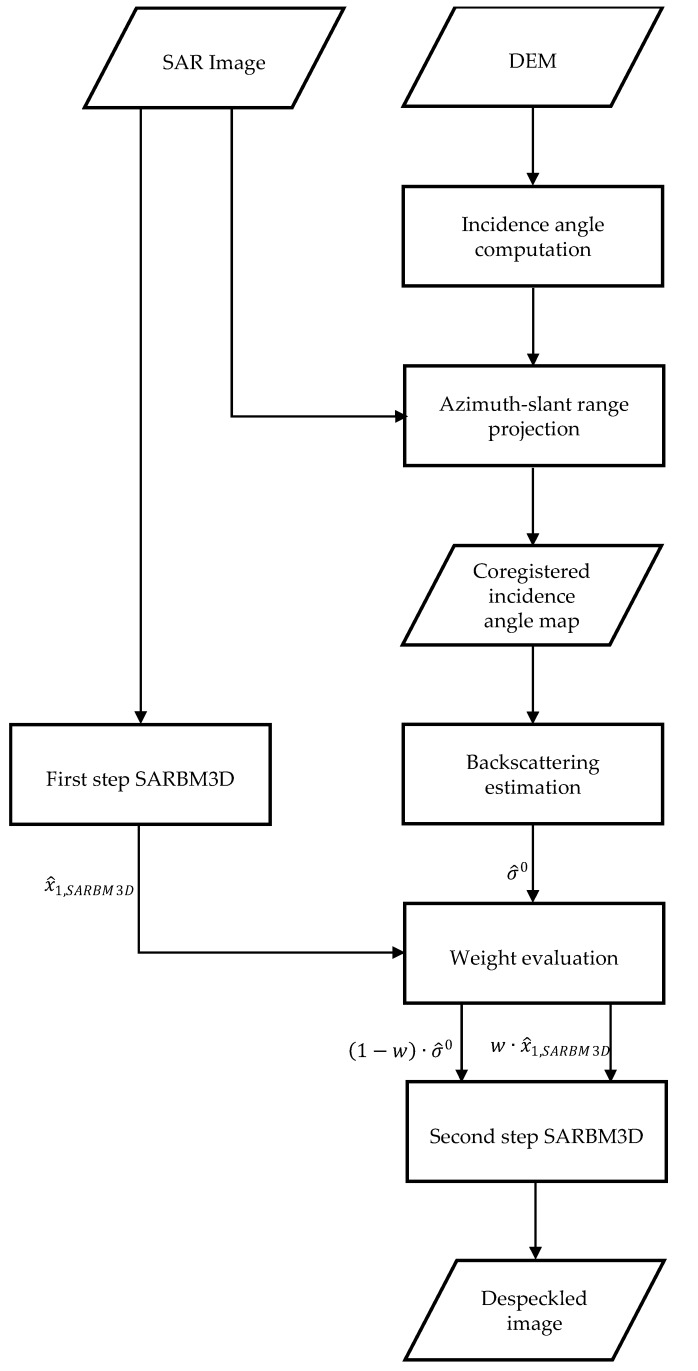
Block scheme of the Scattering-Based (SB)-SAR Block-Matching 3D (SARBM3D) algorithm.

A block scheme of the SB-SARBM3D despeckling algorithm is shown in Figure 1.

## 3. Results

In this section, a comprehensive experimental sensitivity analysis of the SB-SARBM3D despeckling algorithm is carried out, and the main results are discussed. First, in Section 3.1, the influence of the scattering behavior of the surface is analyzed by applying the algorithm to SAR images simulated via different scattering models. Section 3.2 deals with the influence of surface parameters on the despeckling capability of the filter. To this aim, the SB-SARBM3D algorithm is applied to a single-look SAR image with different values for the input surface parameters. Then, in Section 3.3, the role of the spatial resolution of the DEM is investigated and evaluated by applying the algorithm with a priori scattering information estimated from DEMs with different resolutions. Finally, in Section 3.4, the role of coregistration errors between the DEM and the SAR image is analyzed for different DEM resolutions. In Section 3.5, the role of DEM data is assessed also on an actual scenario by applying the SB-SARBM3D algorithm with different resolution DEMs.

**Figure 2:**
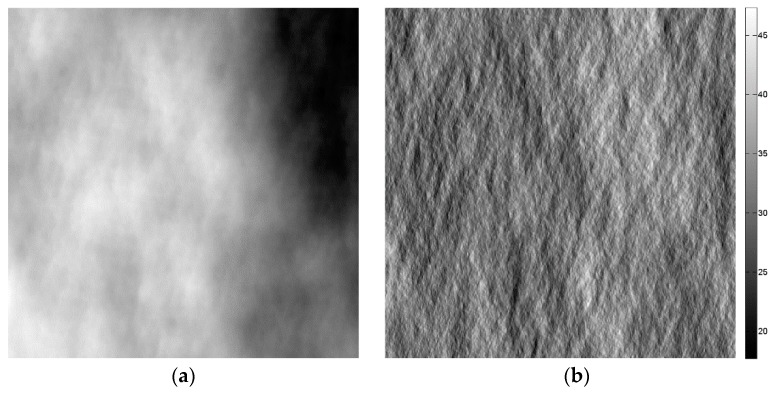

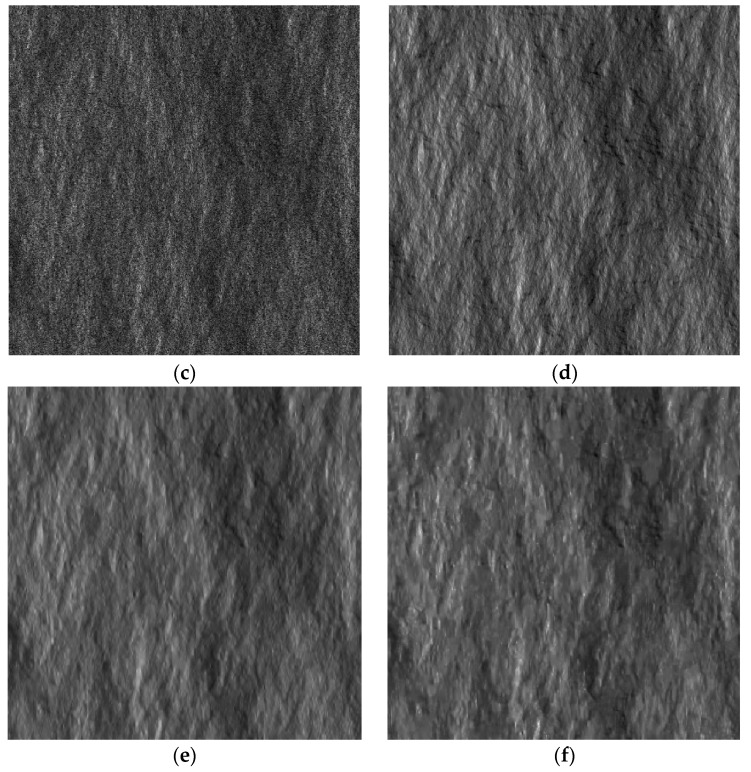
(**a**) Fractal DEM with fractal parameters *H =* 0.8, *T =* 10^−5^ m in the azimuth-slant range coordinate system; the resolution is 2.58 m and 2.29 m in the azimuth and slant range, respectively; (**b**) local incidence angle map in the azimuth-slant range coordinate system; (**c**) 512 × 512 single-look SAR image corresponding to the DEM in (a) and to the electromagnetic parameters *ε_r_ =* 4 and *σ =* 10^−2^ S/m; (**d**) reference image obtained by averaging 512 single-look sample images; (**e**) SB-SARBM3D with a priori scattering information estimated from (b) and assuming the right values for the surface parameters; (**f**) SARBM3D.

For the entire sensitivity analysis, the scene topography is simulated via the 2D fBm surface of fractal parameters H=0.8 and $T=10^{-5}$ m and electromagnetic parameters $\varepsilon_{r}=4$ and $\sigma =10^{-2}\;\text{S}/m$ shown in Figure 2a, while in Figure 2b, the corresponding local incidence angle map is depicted. Otherwise stated, all of the surface parameters, namely, *ϑ*, *H*, *T*, *ε_r_* and *σ*, are assumed to be known in the filter. SAR images are simulated via the SARAS simulator described in [22] with the COSMO-SkyMed sensor parameters [23]. The scattering behavior of the surface is simulated via the SPM option of SARAS, unless otherwise stated. The simulated single-look SAR image corresponding to the DEM in Figure 2a is displayed in Figure 2c.

The despeckling capabilities are quantitatively evaluated via both no-reference and full-reference synthetic parameters. In particular, the Variance of Ratio (VoR), the Coefficient of variation (C_x_), the Signal-to-Noise Ratio (SNR) and the Mean Structural Similarity Index Measure (MSSIM) are computed as described in [24,25]. VoR represents the variance of the ratio between the noisy intensity image and the filtered one. For ideal filtering, the ratio image is pure speckle, and VoR equals one. Therefore, it provides some information about a partial speckle removal (VoR lower than one) and texture smoothing (VoR greater than one). A good texture preservation is also revealed by a coefficient of variation close to the value estimated on the reference image. SNR is a measure of the overall quality of the filtered image: the higher the SNR, the better the overall performance of the filter. A MSSIM value close to one reveals a high structural similarity between the filtered and the reference images. For what concerns SNR and MSSIM, the graphs reported in the following show both the absolute value and the relative value normalized to the maximum. Reference images are computed via the average of 512 sample single-look images. In order to quantitatively establish the quality of the despeckling algorithm, the reader is referred to the reference image measures in Table 1. The reference image corresponding to the SAR image in Figure 2c and to the DEM in Figure 2a is shown in Figure 2d. For a better understanding of the key role of the a priori scattering information, the SB-SARBM3D filter in Figure 2e is also compared to original SARBM3D in Figure 2f.

### 3.1. Sensitivity against the Scattering Behavior of the Surface

**Figure 3:**
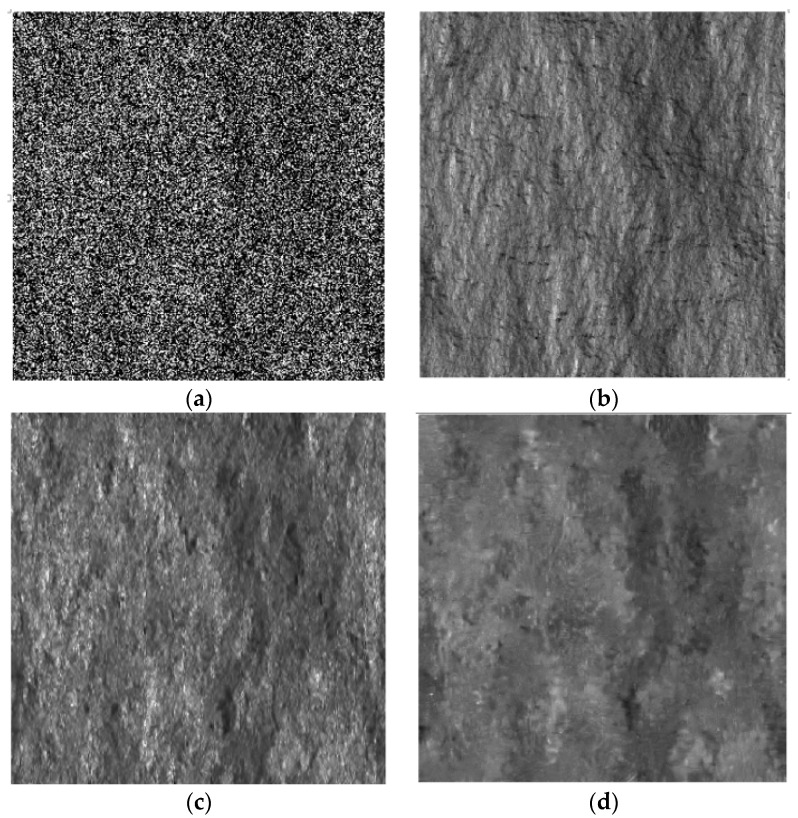
Simulated and despeckled SAR images relevant to the DEM in Figure 2a and assuming the cosϑ scattering model. (**a**) Noisy; (**b**) reference SAR image; (**c**) SB-SARBM3D; (**d**) SARBM3D.

**Figure 4:**
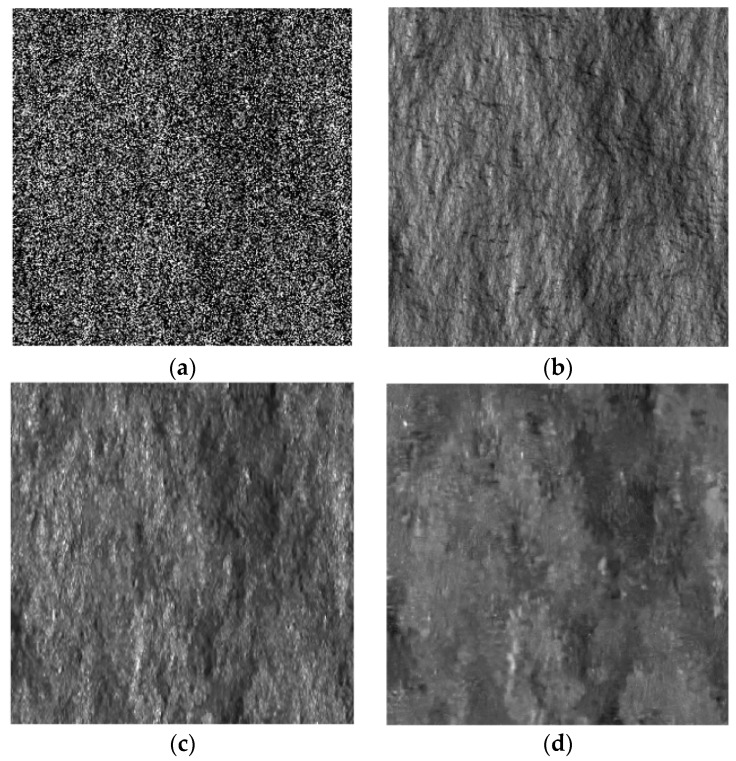
Simulated and despeckled SAR images relevant to the DEM in Figure 2a and assuming the $\cos^{2}\vartheta$ scattering model. (**a**) Noisy; (**b**) reference SAR image; (**c**) SB-SARBM3D; (**d**) SARBM3D.

**Figure 5:**
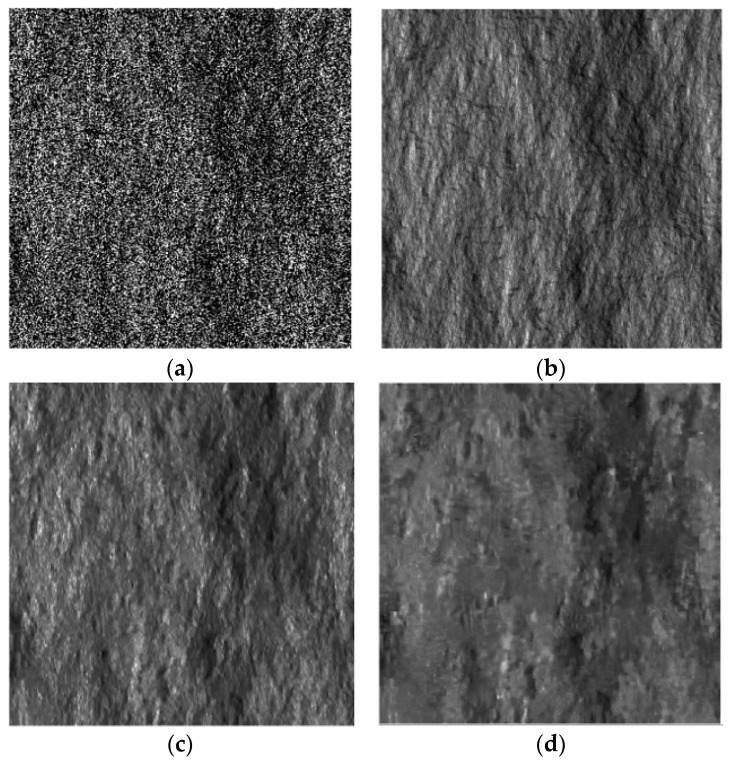
Simulated and despeckled SAR images relevant to the DEM in Figure 2a and assuming the $\cos^{4}\vartheta$ scattering model. (**a**) Noisy; (**b**) reference SAR image; (**c**) SB-SARBM3D; (**d**) SARBM3D.

Several models concerning single-bounce surface scattering have been developed so far. Depending on the surface model used, they can be categorized into “classical” models, in which the surface height is assumed to be normally distributed, and “fractal models”, in which the fractal geometry is used. Following these approaches, the integral equation method, SPM, Physical optics, geometrical optics, Kirchhoff approximation and Generalized Lambertian Law (GLL) were developed and assessed both theoretically and experimentally. For more details, the reader is referred to [17,26,27,28,29]. It is noteworthy that the accuracy of the pilot image in SB-SARBM3D depends on the scattering behavior of the scene. In particular, since the SPM scattering model is assumed within the filter, it is noteworthy that the more accurate the SPM model, the better the results. Since the local incidence angle is primarily exploited within the filter to estimate the backscattering coefficient of the surface, a key point in the sensitivity analysis of the SB-SARBM3D algorithm is the assessment of the filter performance in the presence of surfaces exhibiting different relationships between the local incidence angle and the backscattering coefficient. In order to assess the robustness of the SB-SARBM3D filter against the scattering behavior of the surface, the algorithm is applied to SAR images of the fractal scene previously described, simulated assuming different scattering models. In particular, besides the SPM model, the $\cos\vartheta ,\;{\text{cos}}^{2}\;\vartheta$ and $\cos^{4}\;\vartheta$ GLL scattering models are used for simulation purposes. Single-look SAR images and despeckled images are shown in Figure 2, Figure 3, Figure 4 and Figure 5, while synthetic performance parameters related to both the SB-SARBM3D and SARBM3D filters are reported in Table 2 and Table 3, respectively. SB-SARBM3D exhibits a significant sensitivity against the scattering behavior of the surface. The worst results are provided with the cosϑ, since it is the most dissimilar model to the SPM one. However, it is noticeable that this Lambertian model is not adequate to describe the scattering mechanisms at microwave frequencies, since it states that the energy incident on a surface is equally scattered in any direction. This property is not valid in the operating frequency range typical of SAR systems [17,30,31,32]. Nevertheless, we consider it for its widespread use in some specific applications of SAR imagery, e.g., shape from shading [32,33,34,35,36]. Intermediate performances are provided with $\cos^{2}\;\vartheta$ and $\cos^{4}\;\vartheta$ models, since they describe a scattering behavior more similar to the SPM model than the cosϑ model. However, the exploitation of the a priori scattering information provides a better pilot image w.r.t. SARBM3D, even if the SPM model is not accurate, as shown by the performance improvement over the original SARBM3D filter.

### 3.2. Sensitivity against Surface Parameters

An accurate estimation of the backscattering coefficient of the surface as in Equation (6) requires, at least in principle, an accurate estimation/knowledge of all of the concerned parameters. While the sensor parameters, namely the electromagnetic wavenumber and the radar look angle, are usually provided together with the ancillary data of the image and, therefore, they can be reasonably assumed known, an accurate knowledge of all of the surface parameters, namely the local incidence angle, the Hurst coefficient, the topothesy, the relative dielectric constant and the electrical conductivity, is not realistic, at least where SAR data are of interest. However, the SPM model exhibits a different sensitivity against surface parameters. In particular, as shown in [15] (Appendix), the local incidence angle has the major influence on the energy backscattered from the surface. A key role is also played by the Hurst coefficient, whilst the remaining parameters exhibit a minor influence. However, the SB-SARBM3D algorithm is capable of accounting for the knowledge of whatever surface parameter. For example, in [37], a method to retrieve the soil surface parameters from polarimetric SAR data is presented; in [38], a general framework for surface parameters estimation from backscattered data is described. 

**Figure 6:**
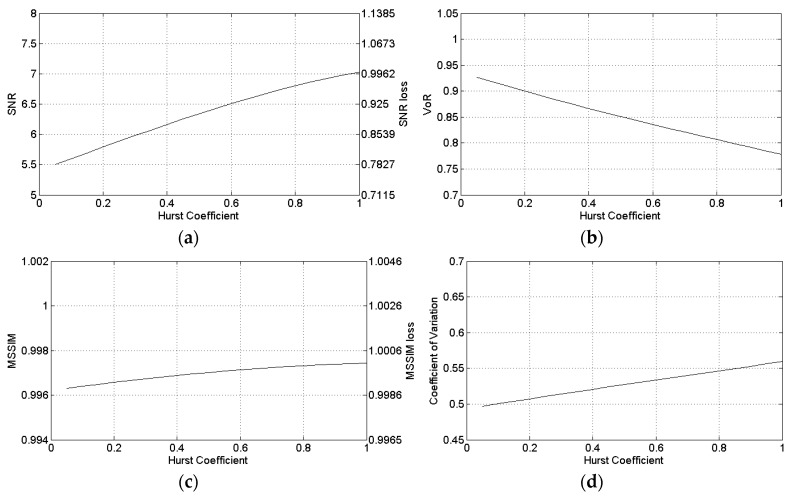
Sensitivity of SB-SARBM3D against the Hurst coefficient: (**a**) SNR; (**b**) VoR; (**c**) MSSIM; (**d**) coefficient of variation.

In this section, the sensitivity of the SB-SARBM3D algorithm against surface parameters is evaluated by means of an experimental analysis. To this aim, the algorithm is applied to the single-look SAR image in Figure 2c and the backscattering coefficient is estimated by using different values of the surface parameters. An accurate knowledge of the local incidence angle, whose key role is investigated further in the paper, is assumed for the a priori scattering information estimation. To assess the sensitivity of SB-SARBM3D against inaccuracy in the Hurst coefficient estimation/knowledge, we apply the algorithm to the single-look SAR image in Figure 2c, relevant to the DEM in Figure 2a, with different values of the input parameter *H*. The performance parameters depicted in Figure 6 show a non-negligible influence of the Hurst coefficient on the filter performance, thus confirming its non-negligible influence on the backscattered energy from the surface [15].

In particular, in this scenario, a performance degradation up to 22% is experienced in correspondence of very gross errors on *H* estimation. However, with typical values of actual natural surfaces (0.6≤H≤0.9) [31], a smaller degradation (up to 8%) is experienced. High *H* values provide less smoothing and a better texture preservation, as witnessed by the VoR and the C_x_ parameters. It is noticeable that the non-negligible influence of the *H* parameter can be faced via a proper estimation procedure, such as that suitable for single-look SAR data proposed in [18]. However, owing to the a priori scattering information, the SB-SARBM3D provides better results w.r.t. the original SARBM3D for every value of *H* (performance parameters of SARBM3D are reported in the SPM row in Table 3). 

**Figure 7:**
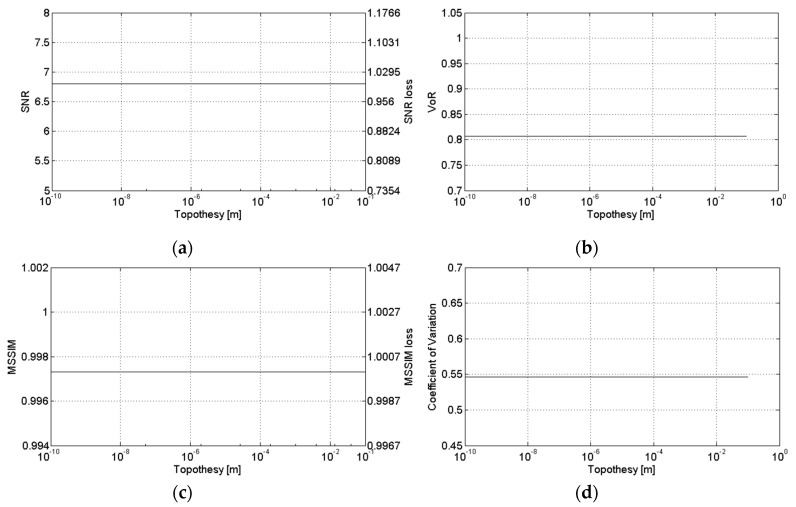
Sensitivity of SB-SARBM3D against the topothesy: (**a**) SNR; (**b**) VoR; (**c**) MSSIM; (**d**) coefficient of variation.

**Figure 8:**
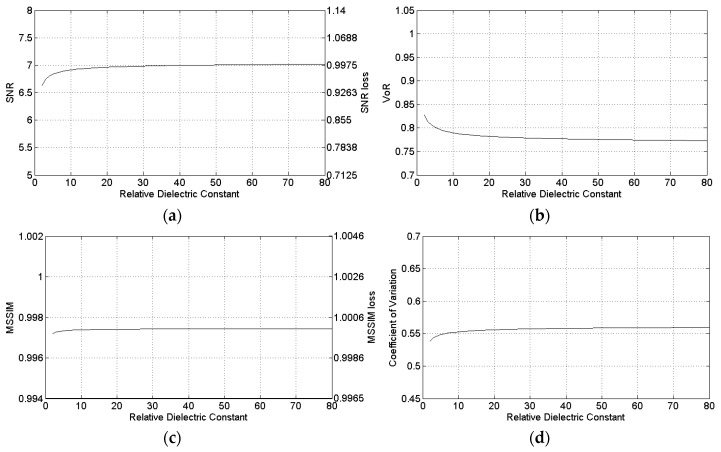
Sensitivity of SB-SARBM3D against the relative dielectric constant: (**a**) SNR; (**b**) VoR; (**c**) MSSIM; (**d**) coefficient of variation.

**Figure 9:**
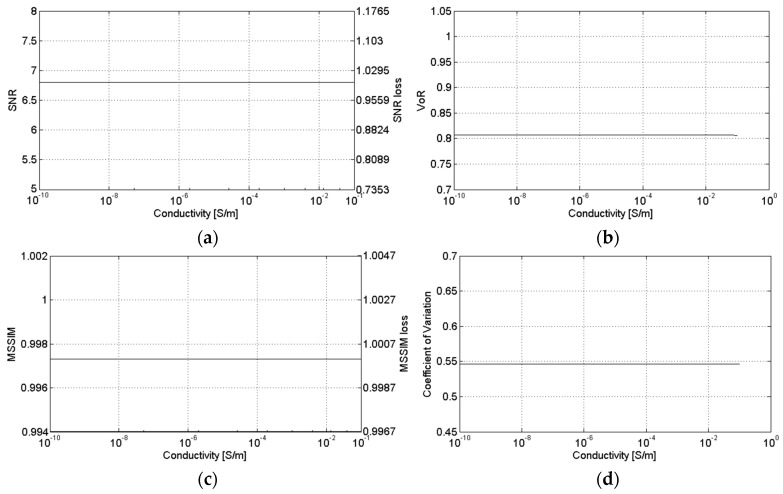
Sensitivity of SB-SARBM3D against the electrical conductivity: (**a**) SNR; (**b**) VoR; (**c**) MSSIM; (**d**) coefficient of variation.

Figure 7, Figure 8 and Figure 9 show the sensitivity of SB-SARBM3D against the topothesy, the relative dielectric constant, and the electrical conductivity, respectively. The minor influence of these parameters on the energy backscattered from the surface reflects itself in the robustness of the despeckling filter, whose performance is negligibly affected by an accurate knowledge of their actual values. Therefore, for such parameters, reference values can be used without incurring a significant performance degradation, if an estimation/knowledge of these parameters is not available.

### 3.3. Sensitivity against the DEM Resolution

In order to apply the SB-SARBM3D filter, a DEM of the scene is required. As previously stated, the ratio maps *r_I_* and *r_ϑ_* are aimed at properly weighing the pilot image provided by the original SARBM3D filter and the a priori scattering information by distinguishing topography-related and non-topography-related SAR intensity variations. It is noticeable that the higher the resolution of the DEM, the higher the probability to correctly detect topographic features.

In this section, the robustness of SB-SARBM3D against the DEM spatial resolution is analyzed by applying the algorithm to the single-look SAR image shown in Figure 2c and by exploiting the a priori scattering information estimated from DEMs with different resolutions. 

The highest-resolution DEM used (Figure 2a) shares the same spatial resolution of the simulated SAR image in Figure 2c, i.e., 2.58 m in azimuth and 2.29 m in slant range.

The high-resolution DEM in Figure 2a is then smoothed via a moving average filter of increasing window size as a power of two up to 512, in order to obtain less detailed DEMs. This low-pass filtering has the advantage of retaining the original grid spacing, while providing a smoothed DEM. Consequently, the filtered DEMs share the same spatial resolution of the SAR image. This is not true when dealing with actual SAR data, since commonly, SAR imagery and DEMs have different spatial spacing. However, the DEM must undergo a projection in the SAR system coordinate in order to apply the SB-SARBM3D filter; this transformation provides a coregistered DEM that shares the spatial resolution of the SAR image via appropriate interpolation methods. An example of a gross DEM is that provided by the Global 30 Arc-Second Elevation (GTOPO30) DEM [39], while DEMs with very high-resolution up to 1 m are provided by LiDAR systems.

**Figure 10:**
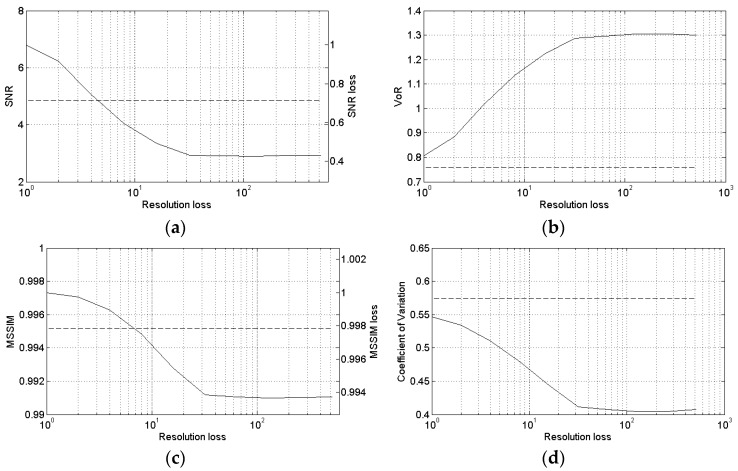
Sensitivity of SB-SARBM3D against the DEM resolution loss: (**a**) SNR; (**b**) VoR; (**c**) MSSIM; (**d**) coefficient of variation. The highest resolution ensures the best performance; SARBM3D (dashed lines).

**Figure 11:**
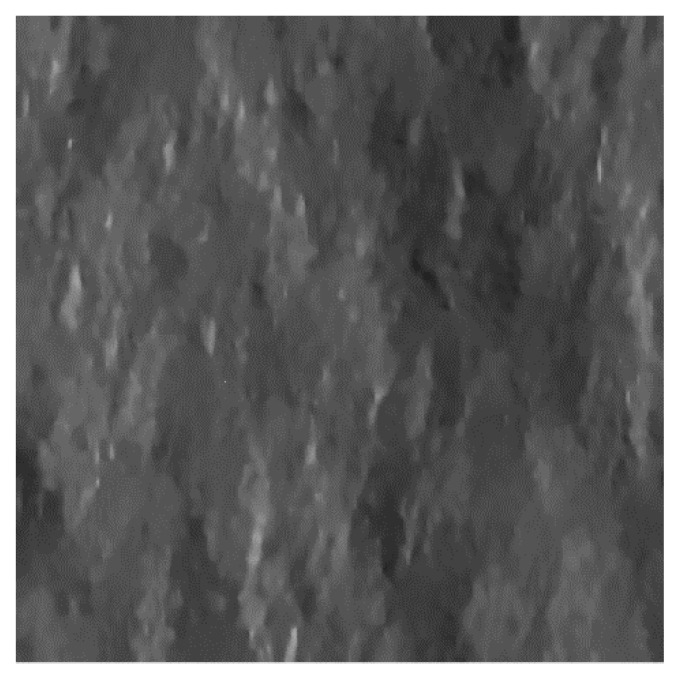
SB-SARBM3D with a priori scattering information estimated from the local incidence angle map in Figure 2b filtered with a 512 × 512 moving average filter and assuming the right values for the surface parameters.

The highest-resolution DEM ensures the best performance, as shown in Figure 10, providing an SNR improvement of more than 40% over the original SARBM3D filter. This is due to the richly detailed a priori scattering information that allows a significant speckle reduction with negligible smoothing effects. The lower the DEM resolution, the smoother the a priori scattering information and the smoother the filtered image. A significant detail loss is visible with the lowest resolutions (Figure 11), as witnessed by the VoR increasing with the DEM spatial spacing. In the considered scenario, with sufficiently low resolution, the a priori scattering information provides worse results than the original SARBM3D filter. Better performance is provided by SB-SARBM3D up to a resolution loss of four, corresponding to a resolution of about 10 m in azimuth and 9 m in slant range in the considered scenario. With lower resolutions, the absence of both non-topographic features and a sufficiently high dynamic range of SAR intensity prevents the assignment of a high weight to the pilot image provided by the SARBM3D; consequently, an increasingly smoothed pilot image is estimated due to the high weight assigned to the a priori scattering term. Therefore, a smoother and smoother despeckled image is obtained with lowering DEM resolutions.

### 3.4. Sensitivity against the DEM Coregistration

In this section, we investigate the role of the coregistration step of SB-SARBM3D. To provide a comprehensive understanding of the analysis, the sensitivity of the algorithm is evaluated for different DEM resolutions. For any DEM resolution, coregistration errors between the DEM and the SAR image are simulated via an increasing displacement of the local incidence angle map in Figure 2b with respect to the SAR image in Figure 2c. 

**Figure 12:**
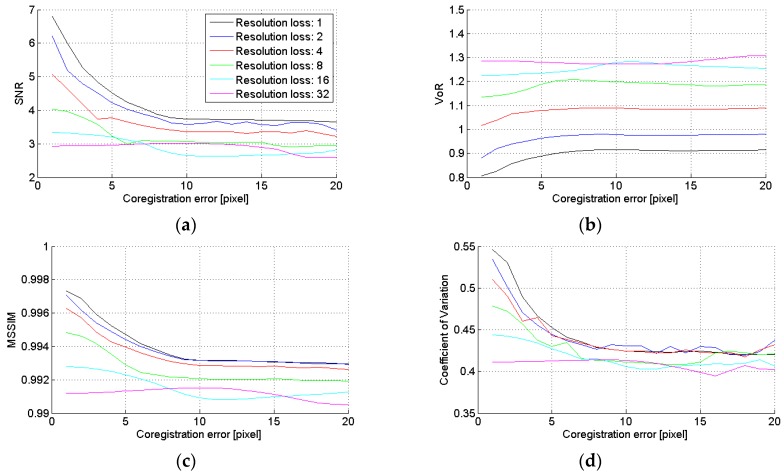
Sensitivity of SB-SARBM3D against coregistration errors (in pixels) between the local incidence angle map and the SAR image for different DEM resolutions. (**a**) SNR; (**b**) VoR; (**c**) MSSIM; (**d**) coefficient of variation. All of the figures share the same legend. Low-resolution DEMs provide smooth a priori scattering information. Consequently, the lower the DEM resolution, the stronger the sensitivity of SB-SARBM3D against coregistration displacements.

For the sake of simplicity, we consider only errors along the range axis. Similar comments apply to (translation/rotation) errors in other directions. The performance parameters shown in Figure 12 indicate that particular attention to the coregistration step should be paid in the presence of a high-resolution DEM. In this case, a significant performance degradation can be experienced if the coregistration step is not accurate. This is due to the significant spatial high-frequency content of the a priori scattering information in the case of high-resolution DEMs. On the contrary, the more homogeneous scattering information estimated from low-resolution DEMs causes a higher robustness of the performance even in the presence of gross coregistration errors. However, with high-resolution DEMs, better performance is provided at the cost of a precise coregistration step. As shown in Figure 12, an accurate coregistration step can compensate a low-resolution DEM, since if a sufficiently high displacement occurs, a high-resolution DEM may provide worse results than a fine-coregistered low-resolution one. In conclusion, the highest resolution DEM should be used, unless the robustness of the filter is of interest. In the latter case, some smoothing of the DEM can be useful to provide less sensitivity against coregistration displacements.

### 3.5. Actual Case Scenario

**Figure 13:**
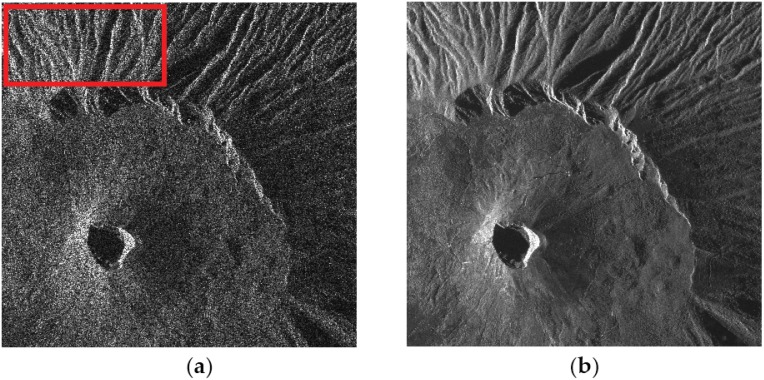

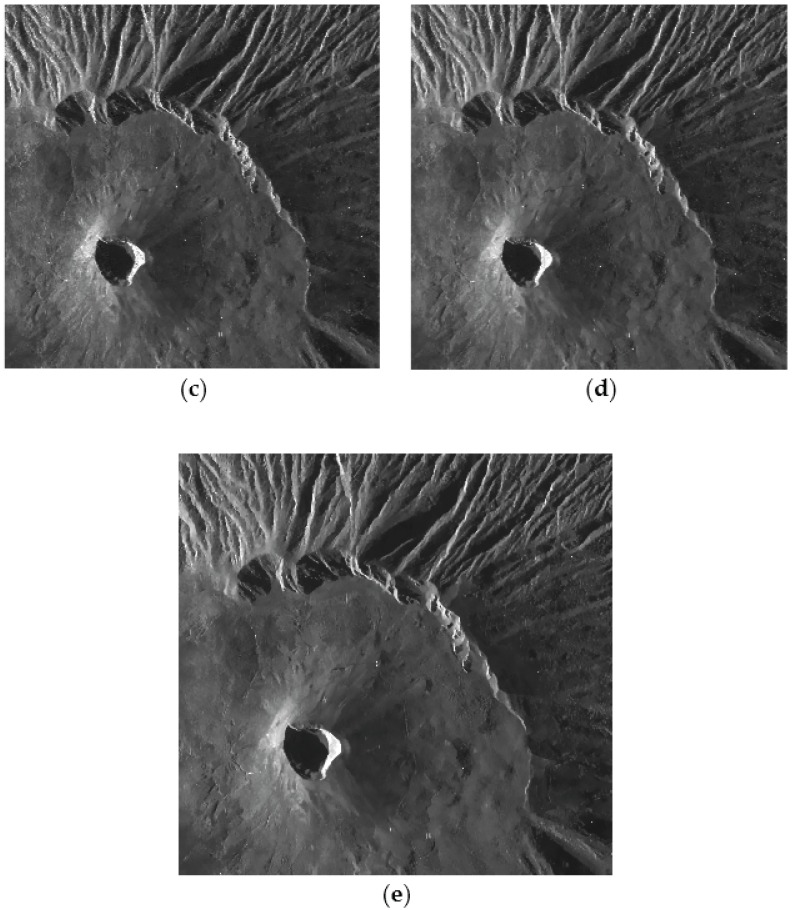
(**a**) 1940×2000 single-look stripmap COSMO-SkyMed SAR image of the Vesuvius-Mt. Somma close to Naples, Italy. Pixel spacing is 2.07 m and 1.18 m in azimuth and slant range, respectively. The red box indicates the region used for the coefficient of variation evaluation. (**b**) 42-look SAR image obtained via temporal multilook and used as the reference. SB-SARBM3D by exploiting a 5-m (**c**), 90-m (**d**), and 1-km (**e**) DEM.

In order to test the key role of the DEM spatial resolution on the filter performance in real-life conditions, the SB-SARBM3D algorithm is applied to a 1940×2000 single-look stripmap COSMO-SkyMed SAR image of the Vesuvius-Mt. Somma close to Naples, Italy (Figure 13a). The radar look-angle is 44°; the operating frequency is 9.6 GHz; and the pixel spacing is 2.07 m and 1.18 m in azimuth and slant range, respectively. A 42-look SAR image obtained via temporal multilook is used as the reference (Figure 13b). The backscattering coefficient is evaluated through Equation (6) by means of the Hurst coefficient estimated via the algorithm proposed in [18] and DEMs with different resolutions. In particular, a 5-m aero-photogrammetric (high-resolution) DEM is filtered with a moving average filter in order to obtain an intermediate-resolution 90-m DEM and a low-resolution 1-km DEM. The SB-SARBM3D outcomes are shown in Figure 13c–e. The DEM resolution causes slight differences among the despeckled images especially in terms of SNR, VoR and the coefficient of variation (Table 4). Despite slightly better overall performance provided with low-resolution DEM data (see the SNR in Table 4), likely caused by an imperfect coregistration, better detail preservation is ensured by a high-resolution DEM: the VoR increases while the coefficient of variation decreases with increasing DEM pixel spacing (see Table 4). A good trade-off between detail preservation and overall performance is provided by the intermediate resolution DEM.

## 4. Conclusions

In this paper, an experimental sensitivity analysis of the recent SB-SARBM3D despeckling filter is conducted. In particular, the influence of the following features on the filter performance is analyzed and discussed:

• scattering model;
• surface parameters errors apart from the local incidence angle;
• DEM resolution (on both simulated and actual SAR images);
• errors in the coregistration step.

Besides the SPM scattering model used in the filter, the GLLs $\cos\vartheta ,{\text{ cos}}^{2}\;\vartheta$ and $\cos^{4}\;\vartheta$ have been used to assess the robustness of the filter against the scattering behavior of the surface. As expected, the best results are provided in the SPM case, whilst the cosϑ causes the worst performance. In general, the better the match between the scattering behavior of the surface and the SPM model, the better the overall performance of the filter. Therefore, intermediate results are provided with the $\cos^{2}\;\vartheta$ and $\cos^{4}\;\vartheta$ models. However, the SB-SARBM3D filter outperforms the original SARBM3D filter for most of the considered scattering models. The research in this field would benefit from a scattering model selection algorithm for a suitable filter model-selection step.

The sensitivity analysis against surface parameters suggests that the huge knowledge required to estimate the a priori scattering information, modeled via the SPM model suitable for natural bare soil surfaces, does not limit the applicability of the filter. Most of the surface parameters, namely topothesy, relative dielectric constant and conductivity, influence very little the energy backscattered from the surface, at least in the presence of a significant topography. Consequently, the accurate knowledge is not strictly required for such parameters, and reference values can be used. As concerns the Hurst coefficient, the retrieval procedure developed in [33] can be exploited in order to deal with its non-negligible influence on the filter performance. However, with typical values, very little performance degradation is experienced, suggesting the use of reference values even for *H* in the case of strict time requirements.

A key role in the despeckling performance, especially concerning the detail preservation capability of the filter, is played by the DEM resolution, as shown with both simulated and actual SAR data. A high-resolution DEM allows for a detailed a priori scattering information estimation. Therefore, the finer the topography details, the better the detail preservation capability of the filter. With low-resolution DEMs, a significant performance drop is experienced, and worse performance than SARBM3D may be provided in the presence of a significantly gross DEM. For DEM resolutions up to a few times the SAR image resolution, better performance is provided by the a priori scattering information. The DEM resolution plays a key role even in the robustness of SB-SARBM3D against coregistration mismatches between the SAR image and the DEM. Thus, a high-resolution DEM, even if providing richly-detailed a priori scattering information, causes a significant performance drop in the presence of coregistration errors, unless the topography is gentle enough. On the contrary, low-resolution DEMs allow a higher robustness of the filter performance against errors in the coregistration step, thanks to the smoother a priori information. It is worth noticing that the feasibility of retrieving the local incidence angle map directly from SAR data is currently under study. This will avoid the need for such extra information and possible coregistration errors.

## Figures and Tables

**Table 1:** Performance parameters of the reference image. VoR, Variance of Ratio; C_x_, Coefficient of variation; MSSIM, Mean Structural Similarity Index Measure; SPM, Small Perturbation Method.

	SNR	VoR	C_x_	MSSIM
SPM	+∞	0.98	0.67	1.00
cos ϑ	+∞	1.01	0.15	1.00
cos^2^ ϑ	+∞	1.02	0.21	1.00
cos^4^ ϑ	+∞	1.01	0.26	1.00

**Table 2:** Performance parameters of SB-SARBM3D.

	SNR	VoR	C_x_	MSSIM
SPM	6.80	0.81	0.55	1.00
cos ϑ	0.93	0.77	0.17	1.00
cos^2^ ϑ	2.19	0.78	0.19	1.00
cos^4^ ϑ	4.02	0.78	0.25	1.00

**Table 3:** Performance parameters of SARBM3D.

	SNR	VoR	C_x_	MSSIM
SPM	4.84	0.76	0.57	0.99
cos ϑ	1.31	0.89	0.11	1.00
cos^2^ ϑ	1.84	0.90	0.14	1.00
cos^4^ ϑ	2.62	0.89	0.19	1.00

**Table 4:** Performance parameters of SB-SARBM3D in the actual case.

	SNR	VoR	C_x_	MSSIM
Reference	+∞	1.00	1.03	1.00
5-m DEM	5.38	1.18	0.93	0.99
90-m DEM	5.67	1.20	0.89	0.99
1-km DEM	5.73	1.34	0.83	0.99
